# Percutaneous closure of large VSD using a home-made fenestrated atrial septal occluder in 18-year-old with pulmonary hypertension

**DOI:** 10.1186/1471-2261-14-74

**Published:** 2014-06-09

**Authors:** Hacer Kamali, Turkay Saritas, Abdullah Erdem, Celal Akdeniz, Volkan Tuzcu

**Affiliations:** 1 Department of Pediatric Cardiology, Istanbul Medipol University, Faculty of Medicine, Istanbul, Turkey; 2 Department of Pediatric and Genetic Arrhythmia, Istanbul Medipol University, Faculty of Medicine, Istanbul, Turkey; 3 Goztepe Mh. Dr. Rifat Pasa Sk. No: 21/33, 34730, Goztepe, Kadikoy, Istanbul, Turkey

**Keywords:** Transcatheter closure, Home-made fenestration, Muscular ventricular septal defect

## Abstract

**Background:**

Hemodynamically significant muscular ventricular septal defects in children after the infantile period are a rare occurrence and ideal for transcatheter closure. In cases of severe concomitant pulmonary hypertension, it may be necessary to fenestrate the device. In this report, we present an 18-year old patient with a large mid-trabecular ventricular septal defect and severe pulmonary hypertension that underwent percutaneous closure of the defect with a home-made fenestrated atrial septal occluder.

**Case presentation:**

An 18-year-old female patient referred to us with complaints of dyspnea (NYHA score of 2–3). Physical examination revealed an apical rumble and a harsh second heart sound. Echocardiographic examination revealed a large mid-trabecular ventricular septal defect with bidirectional shunt and the widest diameter measuring 22 mm on 2D echocardiography. Left and right heart cavities were enlarged. Before and after the vasoreactivity test performed during cardiac catheterization, average aortic pressure was 65 → 86 mmHg, average pulmonary artery pressure: 58 → 73 mmHg, Qp/Qs: 1.6 → 3.2, PVR: 4.6 → 4.3 Wood/U/m^2^ and PVR/SVR: 0.5 → 0.2. On left-ventricular angiocardiogram, the largest end-diastolic defect diameter was 21 mm. The closure procedure was performed with transthoracic echocardiographic guidance, using a 24 mm Cera septal occluder and a 14 F sheath dilator to make a 4.5-5 mm opening. Measured immediately after the procedure and during cardiac catheterization one month later, average aortic pressure was 75 → 75 mmHg, average pulmonary artery pressure: 66 → 30 mmHg, Qp/Qs 1.5 → 1.4, PVR: 4.4 → 2.9 Wood/U/m^2^ and PVR/SVR: 0.4 → 0.2. Transthoracic echocardiographic examination performed 24 hours after the procedure showed a max 35–40 mmHg gradient between the left and right ventricles through the fenestration. After the procedure, we observed sporadic early ventricular systoles and a nodal rhythm disorder that started after approximately 12 hours and spontaneously reverted to normal 9 days later.

**Conclusion:**

In patients with large ventricular septal defects, large atrial septal occluders may be used. In cases with risk of pulmonary vascular disease, a safer option would be to close the defect using a manually fenestrated device.

## Background

Hemodynamically significant muscular ventricular septal defects (VSD) in children after the infantile period are a rare occurrence and ideal for transcatheter closure [1]. Depending on the type, size, location and number of defects, it may be necessary to use an off-label device or multiple devices [2-4]. Additionally, in cases of severe concomitant pulmonary hypertension (PHT), it may be necessary to fenestrate the device [5]. In this report, we present an 18-year old patient with a large mid-trabecular VSD and severe PHT that underwent percutaneous closure of the defect with a home-made fenestrated atrial septal occluder (ASO).

## Case presentation

**Figure 1:**
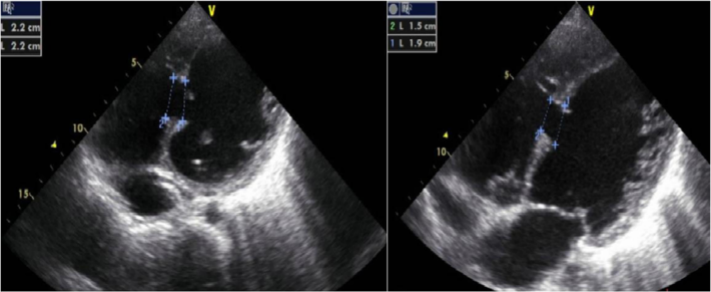
Diameters of VSD on transthoracic echocardiogram.

An 18-year-old female patient referred to us with complaints of dyspnea (NYHA score of 2–3). An apical rumble and a harsh second heart sound were heard in physical examination. Echocardiographic examination revealed a large mid-trabecular VSD with bidirectional shunt and the widest diameter measuring 22 mm on 2D echocardiography (ECHO) (Figure 1; Additional file 1). The defect was located 9 mm proximal to the moderator band. Left and right heart cavities were enlarged; left ventricle, left atrium and right ventricle end-diastolic diameters were respectively 68 mm (Z score: +8.23), 44 mm (Z score: +6.22) and 41 mm (Z score: +10.3). Pulmonary artery diastolic pressure, which we were able to measure due to pulmonary insufficiency, was 58 mmHg. Before and after the vasoreactivity test with inhaled iloprost performed during cardiac catheterization, average aortic pressure was 65 → 86 mmHg, average pulmonary artery pressure: 58 → 73 mmHg, Qp/Qs: 1.6 → 3.2, PVR: 4.6 → 4.3 Wood/U/m^2^ and PVR/SVR: 0.5 → 0.2 (Table 1).

**Table 1:** Hemodynamic parameters during the vasoreactivity test, immediately after the procedure and one month later

	**Before vasoreactivity test**	**After vasoreactivity test**	**Immediately after closure**	**One month later**
**Mean systemic pressure (mmHg)**	65	86	75	75
**Mean PA pressure (mmHg)**	58	73	66	30
**Qp/Qs**	1.6	3.2	1.5	1.4
**PVR ** **(WU/m**^**2**^**)**	4.6	4.3	4.4	2.9
**PVR/SVR**	0.5	0.2	0.4	0.2

PA: Pulmonary artery; Qp/Qs: Pulmonary to systemic flow ratio; PVR: Pulmonary vascular resistance; WU: Wood Unit; PVR/SVR: Pulmonary vascular resistance to systemic vascular resistance ratio.

**Figure 2:**
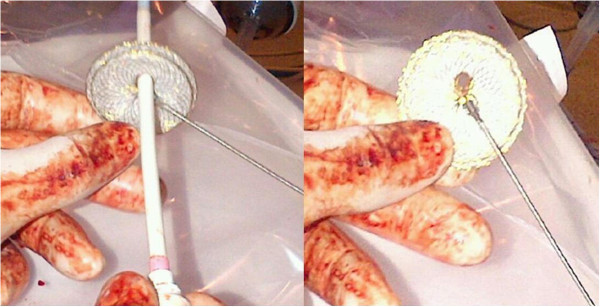
Fenestration procedure and the fenestrated ASD device.

**Figure 3:**
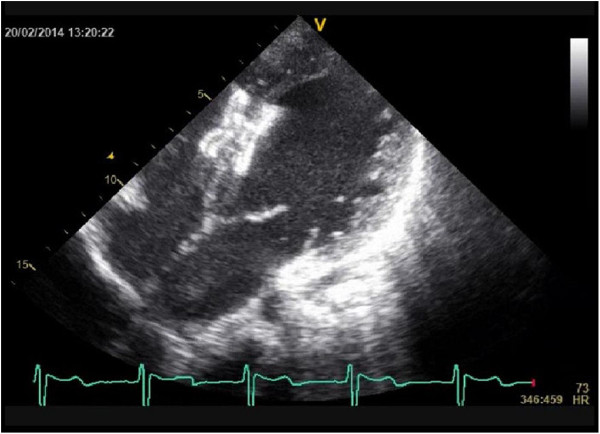
2D imaging of device after the procedure.

On left-ventricular angiocardiogram, the largest end-diastolic defect diameter was 21 mm. The closure procedure was performed with transthoracic echocardiographic guidance, using a 24 mm Cera septal occluder (CSO, Lifetech Scientific Co., ltd, Shenzhen, China) and a 14 F sheath dilator to make a 4.5-5 mm opening (Figures 2 and 3). Measured immediately after the procedure and during cardiac catheterization one month later, average aortic pressure was 75 → 75 mmHg, average pulmonary artery pressure: 66 → 30 mmHg, Qp/Qs 1.5 → 1.4, PVR: 4.4 → 2.9 Wood/U/m^2^ and PVR/SVR: 0.4 → 0.2 (Table 1).

**Figure 4:**
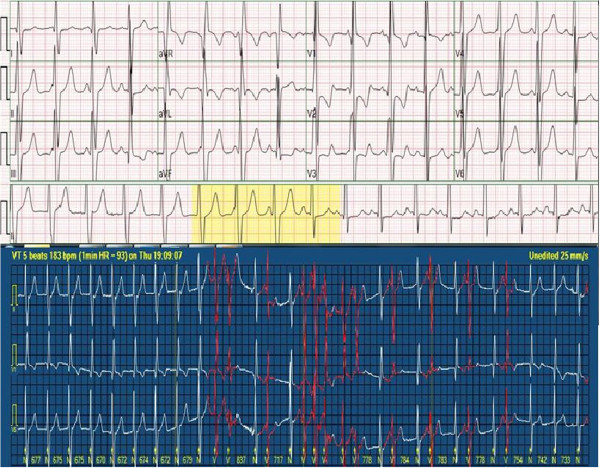
Nodal rhythm (top strip) on ECG and non-sustained VT attack (bottom strip) on 24-hour Holter ECG.

**Figure 5:**
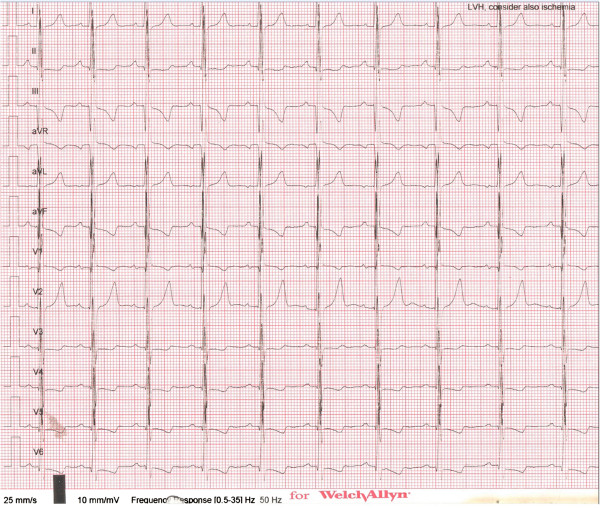
Patient ECG on day 9.

Transthoracic echocardiographic examination performed 24 hours after the procedure showed a max 35–40 mmHg gradient between the left and right ventricles through the fenestration. At the follow-up examination one month later, the gradient level was the same and there was a 50 drop in pulmonary artery diastolic pressure compared to the pre-procedure value.The patient had a normal sinus rhythm prior to the procedure. After the procedure, we observed sporadic early ventricular systoles and a nodal rhythm disorder that started after approximately 12 hours and spontaneously reverted to normal 9 days later. The patient was put on dexamethasone for the nodal rhythm. Propranolol was started after Holter ECG showed intermittent non-sustained ventricular tachycardia (VT) attacks. When the initially intermittent nodal rhythm became permanent, the patient was switched to sotalol. The last ECG and Holter ECG showed a normal sinus rhythm with sporadic early ventricular systoles and no VT attacks (Figures 4 and 5). Holter ECG performed 1 month later showed no early ventricular systoles.

The patient was closely monitored for hemolysis due to the fenestration of the device, but none was observed.

The patient’s dyspnea (NYHA score 2–3 upon admission) started to recede on the 4-5^th^ day after the procedure and was graded as 1–2 in the follow-up examination 1 month later.

## Discussion

Transcatheter closure of congenital and acquired VSDs is a procedure that has gained widespread use in the recent years. Initially, VSD closure was performed using devices designed for atrial septal defects (ASD), but eventually special devices intended for closure of muscular and perimembranous defects were developed [6,7]. Although there has been remarkable progress in production of specifically designed occlusion devices, demand for off-label or custom-made devices suitable for various types of defects remains high [2,3,8].

Transcatheter closure of postinfarction, traumatic or residual postoperative VSDs is mainly performed using ASOs [3,9]. As ECHO and angiocardiographic measurements showed a large defect size in our patient, we elected to use a 24 mm ASO, a device 2–3 mm larger than the defect. The defect could have been closed using a 24 mm muscular VSD occluder or a post-myocardial infarction muscular VSD occluder as well; however, we preferred the ASO as it would allow for more convenient manual fenestration.

Long-term pulmonary hypertension may lead to a fixed increase in pulmonary vascular resistance. In some patients that have undergone VSD closure, pulmonary hypertension may persist or regress very slowly, in which case there may be hypertensive crisis-like exacerbations requiring long-term ventilation and specific treatment. In VSD cases with high pulmonary resistance, post-operative pulmonary hypertensive crises as well as acute congestive heart failure and respiratory failure may prove fatal. For these reasons, surgeons nowadays are instructed to leave an opening in the VSD patch or to use a flap patch that allows for left-to-right shunt [10]. For the same reasons, fenestration is now performed on transcatheter occluder devices. Home-made fenestrated devices have been safely used in cases of ASD with severe PHT under the assumption that fenestrated occluders reduce left-to-right shunt and in the long run, lower pulmonary arterial pressure [5]. Bruch et al. reported using a home-made fenestrated ASO that allowed for a minor to moderate bidirectional shunt in elderly patients with PHT and/or right heart failure. While the authors did not see any deterioration of right or left heart failure in their patient group, they observed an impressive symptomatic benefit [11].

As our patient was an 18-year-old with a large VSD and severe PHT, we performed a vasoreactivity test in order to assess whether the defect could be closed. The patient’s left heart cavities were found to be enlarged, the pulmonary artery flow velocity was high and the response to the vasoreactivity test was positive, so the patient was deemed a suitable candidate for transcatheter closure. However, as the patient was a young adult with bidirectional shunt and an enlarged right ventricle and pulmonary artery, we debated whether or not to fenestrate the device.

A review of literature did not reveal any cases with manually fenestrated devices used for closure of VSDs in patients with severe PHT. However, based on the Bruch study mentioned above and others that used fenestrated ASOs, we assumed that such a device in our case would allow for bidirectional shunt and could possibly reduce PHT in the long run. While no satisfactory reduction in pulmonary artery pressure was seen immediately after the procedure, transthoracic ECHO performed 24 hours later showed left-to-right gradient of 35–40 mmHg through the fenestration.

In the follow-up examination 1 month later, the gradient value was the same, average aortic pressure was 75 mmHg, average pulmonary artery pressure was 30 mmHg and PVR/SVR was 0.2.

Both minor and major complications such as hemolysis, cardiac perforation, device malposition, thromboembolism, temporary or permanent rhythm disorders, endocarditis and death may occur during, immediately after or in the follow-up of transcatheter defect closure [4,12]. In our case, a nodal rhythm started 12 hours after the procedure and spontaneously reverted to normal 9 days later. Temporary junctional rhythms following closure of muscular VSDs have also been reported in the literature [12,13].

Heart block occurring after placement of an oversized device can be attributed to the expansion of the device against the conducting tissue. Even if the device is not oversized, it may cause an inflammatory reaction or formation of scarring in the conduction tissue, in which case steroid therapy may be useful [14]. The nodal rhythm in our patient can be attributed to the fact that an ASO has a larger left disc and a narrower gap between the discs than a VSD device, which may have put pressure on the conduction system or the neighboring tissues.

## Conclusion

In patients with large VSDs, large ASDs may be used as well as large muscular VSD occluders or post-myocardial infarction muscular VSD occluders. In cases with risk of pulmonary vascular disease, a safer option would be to close the defect using a manually fenestrated device.

## Consent

Written informed consent was obtained from the patient for publication of this case report and any accompanying images. A copy of the written consent is available for review by the Editor of this journal.

## Abbreviations

VSD: Ventricular septal defects; PHT: Pulmonary hypertension; ASO: Atrial septal occluder; ECHO: Echocardiography; VT: Ventricular tachycardia; ASD: Atrial septal defects.

## Competing interests

The authors declare that they have no financial and/or non-financial competing interests.

## Authors’ contributions

AE and TS performed the transcatheter intervention. HK gathered patient data. CA and VT treated the patient’s arrhythmia, and contributed to the conception of the report. TS wrote the report. AE checked the report in terms of English grammar. All authors gave their final acceptance to the submission of this report. All authors read and approved the final manuscript.

## Pre-publication history

The pre-publication history for this paper can be accessed here:

http://www.biomedcentral.com/1471-2261/14/74/prepub

## Supplementary Material

Additional file 1 2D and color flow echocardiographic imaging of VSD. Click here for file
